# Pharmacological suppression of glycogen synthase kinase-3 reactivates HIV-1 from latency via activating Wnt/β-catenin/TCF1 axis in CD4^+^ T cells

**DOI:** 10.1080/22221751.2022.2026198

**Published:** 2022-02-01

**Authors:** Jing Wen, Xin Li, Qing-Xia Zhao, Xiao-Fan Yang, Meng-Li Wu, Qihong Yan, Junbiao Chang, Haikun Wang, Xia Jin, Xiao Su, Kai Deng, Ling Chen, Jian-Hua Wang

**Affiliations:** a Institut Pasteur of Shanghai, Chinese Academy of Sciences, Shanghai, People’s Republic of China; b University of Chinese Academy of Sciences, Beijing, People’s Republic of China; c Guangzhou Institutes of Biomedicine and Health, Chinese Academy of Sciences, Guangzhou, People’s Republic of China; d Department of Infection, Zhengzhou Sixth People's Hospital, Zhengzhou, People’s Republic of China; e Institute of Human Virology, Key Laboratory of Tropical Disease Control of Ministry of Education, Zhongshan School of Medicine, Sun Yat-sen University, Guangzhou, People’s Republic of China; f College of Life Science, Henan Normal University, Xinxiang, People’s Republic of China

**Keywords:** HIV-1, viral latency, glycogen synthase kinase-3, β-catenin, TCF1

## Abstract

HIV-1 latency posts a major obstacle for HIV-1 eradication. Currently, no desirable latency reversing agents (LRAs) have been implicated in the “Shock and Kill” strategy to mobilize the latently infected cells to be susceptible for clearance by immune responses. Identification of key cellular pathways that modulate HIV-1 latency helps to develop efficient LRAs. In this study, we demonstrate that the Wnt downstream β-catenin/TCF1 pathway is a crucial modulator for HIV-1 latency. The pharmacological activation of the β-catenin/TCF1 pathway with glycogen synthase kinase-3 (GSK3) inhibitors promoted transcription of HIV-1 proviral DNA and reactivated latency in CD4^+^ T cells; the GSK3 kinase inhibitor 6-bromoindirubin-3′-oxime (6-BIO)-induced HIV-1 reactivation was subsequently confirmed in resting CD4^+^ T cells from cART-suppressed patients and SIV-infected rhesus macaques. These findings advance our understanding of the mechanisms responsible for viral latency, and provide the potent LRA that can be further used in conjunction of immunotherapies to eradicate viral reservoirs.

## GRAPHICAL ABSTRACT

**Figure:**
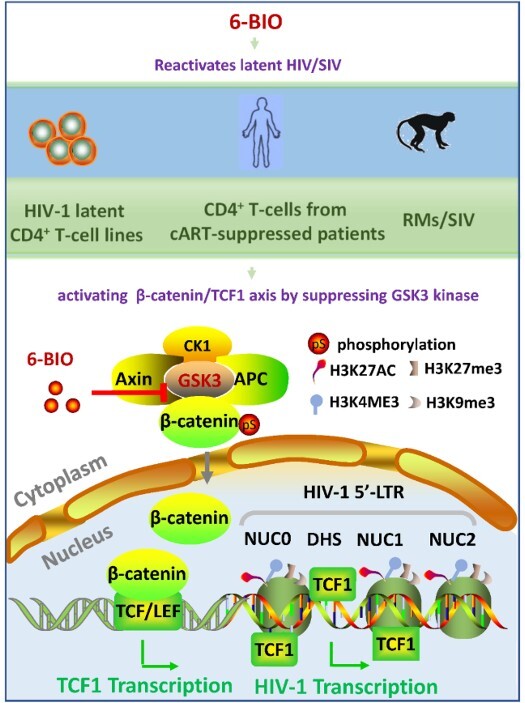

## Introduction

The combination antiretroviral therapy (cART) has changed AIDS from a fatal disease into a chronic disease. However, the persistence of latent viral reservoirs posts an obstacle for cure [1–3]. The latent reservoir is described as a hidden sanctuary of the HIV-1 that is not recognized by the immune system, and the life-long cART is necessary to prevent viral rebound [1–3]. The “Shock and Kill” paradigm represents a potential therapeutic approach to purge HIV-1 reservoirs, in which HIV-1 provirus expression in latently infected cells was reactivated with latency reversing agents (LRAs) (“Shock”), followed by immune responses or ART that clear the activated cells (“Kill”) [1–3]. Although dozens of LRAs with distinct mechanistic classes have been tested in clinical trials, no LRA that fully induce viral reactivation has been identified, this is partly due to the heterogeneity of cells and tissues that form the HIV-1 reservoirs and the complexity of molecular mechanisms that regulate viral latency [4–8]. The low inducibility of latent proviruses is a major problem for the “Shock and Kill” strategy for curing HIV-1 infection [9]. Recently, the administration of compound AZD5582 for activating the non-canonical NF-κB pathway or the interleukin-15 superagonist N-803 in conjunction with CD8^+^ lymphocyte depletion has shown the potent reactivation of HIV or SIV in infected humanized mice and rhesus macaques, respectively [10,11]. Identification of the key host factors/pathways that modulate viral latency helps develop new antiretroviral therapies.

Glycogen synthase kinase-3 (GSK3) is a ubiquitously expressed, highly conserved serine/threonine protein kinase found in all eukaryotes [12]. GSK3 is constitutively active in most cells and serves as a regulator for multiple biological processes, ranging from glycogen metabolism, cell development, gene transcription, and protein translation to cytoskeletal organization, cell cycle regulation, proliferation, and apoptosis [12]. Accordingly, the aberrancy in the GSK-3 activity has been implicated in a wide variety of diseases; thus, targeting GSK3 as a therapy has been tested clinically in human diseases. These include GSK3 inhibitors such as Tideglusib, LY2090314, Enzastaurin, and LiCl [13–15], though the potential off-target pharmacological and toxicological effects necessitate extensive evaluation for their usage [16,17].

GSK3 is a key negative regulator of canonical Wnt signalling, and in the turned-off status of Wnt signalling, GSK3 functionally associates with axin and adenomatous polyposis coli (APC) protein to phosphorylate β-catenin for the sequential proteasomal degradation; while the activation of Wnt signalling prevents GSK3-mediated β-catenin phosphorylation and stimulates the subsequent translocation of β-catenin to nucleus for regulating gene transcription [18–20]. The nuclear-located β-catenin binds factors of the T-cell factor/lymphoid enhancer-binding factor (TCF/LEF) family, enabling the recognition of Wnt target genes through TCF/LEF-specific sequence motifs [18–20].

In this study, we found that pharmacological suppression of GSK3 by small compound 6-BIO reactivated HIV-1 from latently infected CD4^+^ T cells, and 6-BIO-induced HIV-1 reactivation was evaluated in SIV-infected rhesus macaques and Peripheral blood mononuclear cells (PBMCs) isolated from cART-suppressed patients.

## Materials and methods

### Ethics statement

The usages of human samples and rhesus macaques (*Macaca mulatta*) and the related methods and experimental protocols have been approved by the Medical Ethics Review Committee of Institut Pasteur of Shanghai, Chinese Academy of Sciences (CAS), the Medical Ethics Review Committee of Zhengzhou Sixth People's Hospital, and the Institutional Animal Care and Use Committee of Guangzhou Institutes of Biomedicine and Health, CAS. Informed consent has been signed by HIV-1 patients. All experiments were performed in accordance with relevant national guidelines and regulations.

### Cells and virus

The HIV-1 latently infected CD4^+^ CEM cell ACH2 and HIV-1 chronically infected U937 cells (U1) were provided by Dr Shibo Jiang and Dr Lu Lu (Fudan University, Shanghai, China). HeLa-cell-derived TZM-bl indicator cells which contain LTR-driven luciferase reporter were a gift from Dr Paul Zhou (Institut Pasteur of Shanghai, Chinese Academy of Sciences, Shanghai, China). The HEK293T cells were kindly provided by Dr Li Wu (The Ohio State University, USA). Cells were cultured in RPMI 1640 medium (Gibco) or Dulbecco's Modified Eagle Medium (DMEM) (Hyclone) supplemented with 10% fetal bovine serum (Gibco), 100 U/ml penicillin, and 100 μg/ml of streptomycin (Invitrogen) at 37°C under 5% CO_2_.

For assessing HIV-1 reactivation from cells of ACH2, Jat-10.6 and Jat-A2 cells (1 × 10^6^) were stimulated with different concentrations of 6-bromoindirubin-3′-oxime (6-BIO) (T1917; Topscience) (200, 500 nM, 1 μM), or lithium chloride (LiCl) (A100416-0025; Sangon Biotech) (25 mM) for 24 h. Viral reactivation was detected by quantifying the cell-associated *gag* mRNA or titrating the produced viral particles in TZM-bl indicator cells by measuring luciferase activity. Viral reactivation in J-Lat 10.6 and A2 was detected by measuring GFP expression [21].

Pseudotyped single-cycle infectious HIV-Luc/NL-3 was harvested from the co-transfection of HEK293 T cells with the luciferase reporter HIV-1 proviral plasmid pLAI-Δ-env-Luc and the expression plasmid HIV-1-NL4-3 Env (CXCR4 tropic).

### shRNAs

The targeted sequences of shRNAs were listed in Supplementary Table 1. shRNA was cloned into the PLKO.1-puro shRNA expression vector. The packaging of shRNA lentiviruses was performed according to the PLKO.1 protocol (Addgene). Calcium phosphate-mediated transfection of HEK293T cells was used to generate shRNA lentiviruses as previously described [22].

### Real-time qPCR

Total cellular RNA was extracted by TRIzol reagent (Invitrogen), and reverse transcribed into cDNA using the ReverTra Ace qPCR reverse transcription master mix with gDNA (genomic DNA) Remover (Toyobo). Real-time polymerase chain reaction (PCR) was performed using the Thunderbird SYBR qPCR Mix (Toyobo) with pre-denaturation at 95°C for 2 min, amplification with 40 cycles of denaturation (95°C, 15 s), and annealing (60°C, 30 s), on the ABI 7900HT Real-Time PCR system. The data were analysed by a SYBR green-based system (Toyobo), semi-quantified and normalized with glyceraldehyde-3-phosphate dehydrogenase (GAPDH). The primers were listed in Supplementary Table 1. Initial primers are targeted base pair 10–59 of the HIV-1 transcript; Proximal (Pro) primers are targeted base pairs 29–180 of the HIV-1 transcript; Intermediate (Int) primers are targeted base pair 836–1015 of the HIV-1 transcript; Distal (Dis) primers are targeted base pair 2341–2433 of HIV-1 transcript.

### Antibodies and immunoblotting

The following antibodies were used for Chromatin Immunoprecipitation (ChIP) or immunoblotting: anti-TCF1(2003S; Cell Signalling Technology), anti-beta-catenin (ab32572; Abcam), anti-Phospho-β-Catenin (9561s; Cell Signalling Technology), anti-GSK3(ab40870; Abcam), anti-GSK3 phospho Y216 + Y279(ab68476; Abcam), anti-H3K4me3 (9751s; Cell Signalling Technology), anti-H3K27ac (8173s; Cell Signalling Technology), anti-H3K27me3 (17–622, Millipore), anti-H3K9me3 (ab8898, Abcam), anti-GAPDH (M20006; Abmart), and anti-Lamin B (66091-1-Ig, Proteintech).

For immunoblotting, cells were lysed for 1 h at 4°C in ice-cold lysis buffer (50 mM HEPES, pH 7.4, 150 mM NaCl, 0.5 mM EGTA, 1% protease inhibitor cocktail [Sigma], 1 mM sodium orthovanadate, 1 mM NaF, 1% [vol/vol] Triton X-100, and 10% [vol/vol] glycerol). After centrifugation for 10 min at 12,000*g*, the supernatant was boiled in reducing SDS sample loading buffer and subjected by SDS-PAGE. The indicated specific primary antibodies were used, followed by horseradish peroxidase-conjugated goat anti-mouse IgG or goat anti-rabbit IgG (Sigma) as the secondary antibody. Nuclear and cytoplasmic protein fractions were purified using NE-PER Nuclear and Cytoplasmic Extraction Reagents (Thermo Scientific) according to the manufacturer’s introduction.

### Chromatin immunoprecipitation

ChIP experiments were performed according to the protocol provided by EZ-ChIP Chromatin Immunoprecipitation kit (Millipore) as previously described [22]. Cells were cross-linked with 1% formaldehyde for 10 min at room temperature and quenched with 0.125 M glycine for 5 min. After lysis, chromatin was sheared by use of a sonicator (Bioruptor UCD-200; Diagenode) for a total of 12 min (2s on and 6s off) on ice to obtain DNA fragments of 200–500 base pairs. Five per cent of the total sheared chromatin DNA was used as the input sample. Other sheared chromatin was incubated overnight at 4°C with an antibody against TCF1, H3K4me3, H3K27ac, H3K9me3, H3K27me3, or rabbit IgG (Cell Signalling), followed by incubating with 40 μl Protein G/A-labelled Dynabeads at 4°C for 4 h for immunoprecipitation. After washing and reversing cross-link, the input and immunoprecipitated DNA was purified and analysed by real-time PCR using primers specifically targeting for nucleotide position of HIV-1 proviral DNA. The primers were listed in Supplementary Table 1.

### Viral reactivation in resting CD4^+^ T cells from cART-suppressed HIV-1 infected patients

Peripheral blood from combination antiretroviral treatment (cART)-treated HIV-1 infected patients were collected from the cohort patients of Zhengzhou Sixth People's Hospital. Informed consent has been signed. These patients had undergone cART comprised of TDF (tenofovir disoproxil fumarate), 3TC (lamivudine), and EFV (efavirenz) for more than 3 years with an undetectable HIV-1 viral load in the plasma. CD4^+^ counts were analysed by flow cytometry (BD Biosciences). HIV-1 viral loads were quantified by PCR (Cobas Amplicor). HIV subtypes were determined by phylogenetic analysis of Gag-RT sequence with subtype reference (Supplementary Table 2).

PBMCs from cART-treated HIV-1 infected patients were collected with Ficoll-Paque density gradient centrifugation (GE Healthcare Life Sciences). PBMCs were treated with 6-BIO (T1917; Topscience) (1 μM) or phytohemagglutinin-P (PHA-P) (Sigma-Aldrich) (5 μg/mL) for 5 days and cultured in RPMI 1640 medium (Gibco) supplemented with 10% foetal bovine serum (Gibco) in the presence of recombinant interleukin-2 (IL-2) (RD Systems). HIV-1 reactivation was monitored by detecting the surface expression of HIV Envelope (Env) and the production of *gag* and *tat*-*rev* mRNA. Anti-CD3 PerCP-Cy5.5 (eBioscience), anti-CD4 PE-cy7 (BD Biosciences), N6 (anti-gp120 broadly neutralizing antibody), and PE-streptavidin (BD Biosciences) were used for immunostaining. N6 antibody was provided by Jing-He Huang (Fudan University, Shanghai, China). Env positive cells were detected by Fortessa flow cytometer (BD Pharmingen) and analysed with the assistance of FlowJo 7.6.1 software.

### Rhesus macaque performances

Chinese rhesus macaques (*Macaca mulatta*) were housed in the Experimental Animal Center of Guangzhou Institutes of Biomedicine and Health (Guangzhou, China). SIV_mac239_ (5, 000TCID50) intravenously challenged macaques were treated with a potent and long-lasting nucleoside reverse transcriptase inhibitor FNC (2’-deoxy-2’-β-fluoro-4’-azidocytidine) (0.4 mg/kg) we developed recently for viral suppression (Chang J et al., patents: ZL 200710137548.0, US 8835615 B2, EP2177527 B1) [23]. Animals were followed intravenously treated with 6-BIO (T1917; Topscience). Levels of plasma and cell-associated SIV *gag* mRNA were quantitated by real-time PCR as described previously [24]. Briefly, plasma viral RNA was isolated using the QIAamp Viral RNA Minikit (Qiagen) and cellular RNA was extracted using a Total RNA Extraction Kit (Promega). RNAs were reverse transcribed into complementary DNA (cDNA) using the ReverTra Ace qPCR reverse transcription master mix with gDNA (genomic DNA) Remover (Toyobo). Plasma viral RNA copy number was then determined with a SYBR green-based real-time quantitative PCR (Takara) using SIV-*gag*-specific primers (Supplementary Table 1). The copy number of viral RNA was calculated based on the standard curve of an in vitro-transcribed fragment of the SIVmac239 *gag* gene. The limitation for assay was 100 copies/mL plasma. The reverse transcribed cellular cDNAs were used as the template for PCR amplification by Phanta Max Super-Fidelity DNA Polymerase (Vazyme) with SIV-*gag*-specific 1st pair of primers (Supplementary Table 1); the diluted PCR products were further used as the qPCR template, and cell-associated *gag* mRNA number was determined by real-time qPCR with TB Green Premix Ex Taq II (TAKARA) with SIV-gag-specific 2nd pair of primers (Supplementary Table 1). The number of circulating CD3^+^, CD4^+^, and CD8^+^ T-lymphocytes were determined using BD TruCount tubes according to the manufacturer's instructions (BD Biosciences).

### RNA sequencing and data analysis

Total RNAs from cells were extracted using Trizol (Invitrogen) according to the manufacturer’s protocol, and ribosomal RNA removed using QIAseq FastSelect-rRNA HMR Kits (QIAGEN, Germany). Fragmented RNAs (average length approximately 200 bp) were subjected to first strand and second strand cDNA synthesis, followed by adaptor ligation and enrichment with a low-cycle according to the instructions of NEBNext UltraTM RNA Library Prep Kit for Illumina (NEB, USA). The purified library products were evaluated using the Agilent 2200 TapeStation and Qubit2.0 (Life Technologies, USA). The libraries were paired-end sequenced (PE150, Sequencing reads were 150 bp) using Illumina HiSeq 3000 platform.

Raw RNA sequencing (RNA-seq) reads were filtered using Trimmomatic v0.36. The filtered reads were mapped to the human (hg38) reference genomes using HISAT v2.1 with corresponding gene annotations (GRCh38.p13) with default settings. Total counts per mapped gene were determined using *featureCounts* function in SubReads package v1.5.3 with default parameter. Next, counts matrix obtained from *featureCounts* was used as input for differential expression gene analysis with the bioconductor package DESeq2 v1.26 in Rv4.0. Gene counts more than 5 reads in a single sample or more than 50 total reads across all samples were retained for further analysis. Filtered counts matrix was normalized using the DESeq2 method to remove the library-specific artefacts. Principal component analysis was based on global transcriptome data using the build-in function *prcomp* in R software. The genes with log2 fold change >1 or < −1 and adjusted *p* value <0.05 corrected for multiple testing using the Benjamini-Hochberg method were considered significant. Transcription-factor enrichment analysis and functional enrichment analysis were performed using Metascape server tool (https://metascape.org/gp/index.html#/main/step1). Gene set enrichment analysis (GSEA) used the R package clusterProfiler v3.18.1.

### Statistical analysis

Graphpad Prism 8.0 was used for statistical analysis. For intra-group direct comparisons, Student’s unpaired two-tailed t test was performed to analyse significant differences. For comparisons of multiple groups, one-way ANOVAs were performed. Significance levels are indicated as * *p* < 0.05, ** *p* < 0.01, *** *p* < 0.001.

### Data and code availability

All raw RNA-seq data used in this study are available under BioProject accession code PRJNA747246 at the NCBI database (https://www.ncbi.nlm.nih.gov/bioproject/PRJNA747246).

## Results

### Suppression of GSK3 kinase activity by 6-BIO reactivates HIV-1 from multiple latently infected CD4^+^ T cells

**Figure 1.**
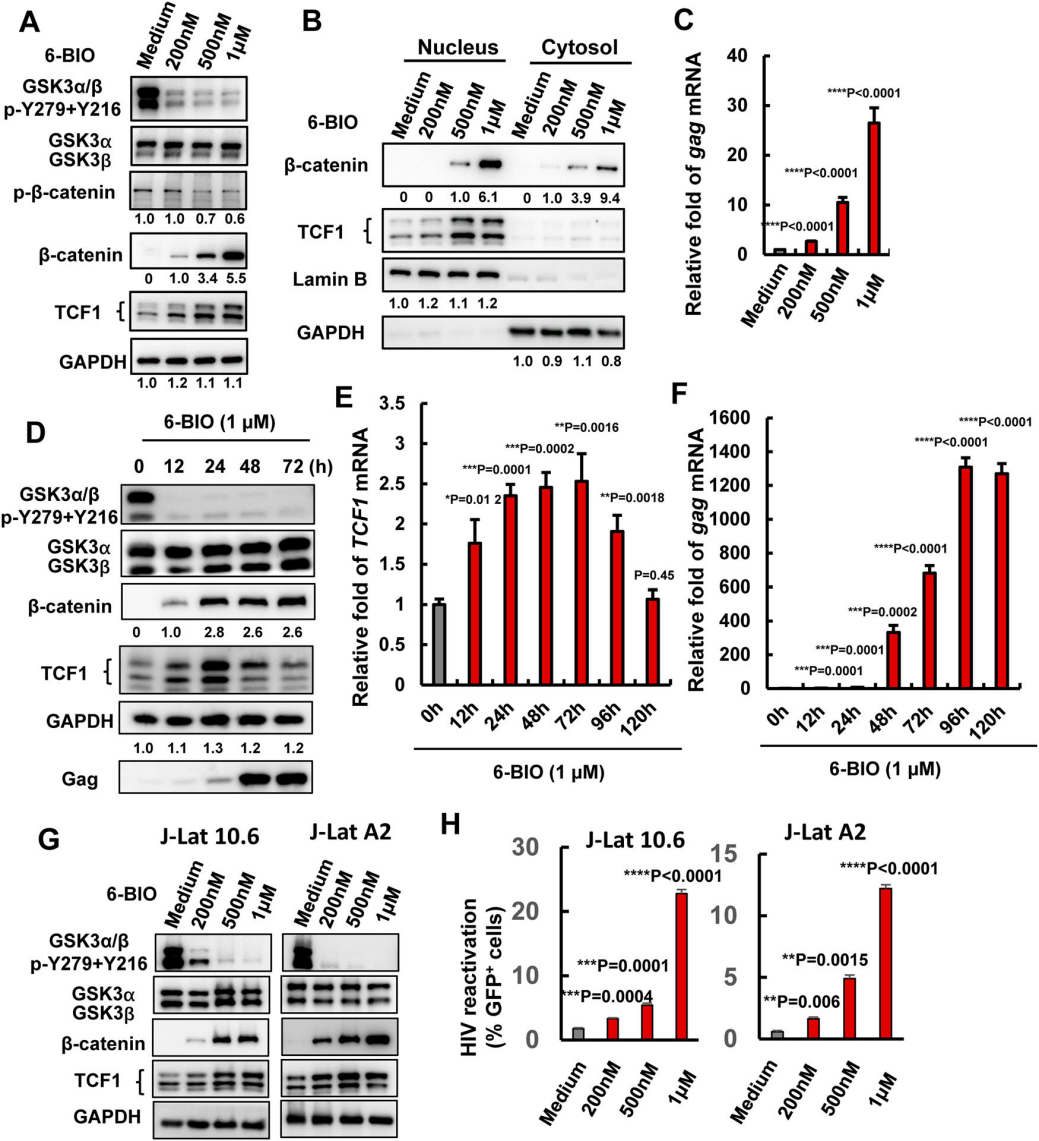
Suppression of GSK3 kinase activity by 6-BIO reactivates HIV-1 from CD4^+^ T cells. ACH2 cells were incubated with indicated concentrations of 6-BIO for 24 h, (A) the expressions of GSK3, β-catenin and TCF1 and their phosphorylated forms were detected by Western blotting with specific antibodies; (B) the nuclear translocation of β-catenin and TCF1 were detected by Western blotting; (C) HIV-1 reactivation was measured by quantifying the production of *gag* mRNA. (D–F) Assay for GSK3 inhibition, TCF1 expression, and HIV-1 reactivation. (G, H) 6-BIO inhibits GSK3 activity and reactivates HIV-1 in J-Lat cells. J-Lat 10.6 or J-Lat A2 cells were incubated with indicated concentrations of 6-BIO for 24 h, and the inhibition of GSK3 activity and the expressions of β-catenin and TCF1 were detected by Western blotting (G), and viral reactivation was detected by quantifying the GFP^+^ cells (H). Grey intensity of the Western blotting strips was analysed with software of Image J (A, B, D). Result is one representative from five independent repeats. Data are presented as mean ± SD. **P < 0.01, *** *P* < 0.001, and ****P < 0.0001 denote significant difference.

To investigate the role of GSK3 in modulating HIV-1 latency, GSK3 inhibitor 6-BIO was used to treat the HIV-1 latently infected CD4^+^ T cell ACH2, and viral reactivation was investigated. GSK3 exists as two isoforms encoded by separate genes: GSK3α (51 kDa) and GSK3β (47 kDa). The treatment of ACH2 cells with 6-BIO inhibited GSK3 kinase activity as demonstrated by the diminished phosphorylation of both α and β isoforms of GSK3 at Tyr216 and Tyr 279 (Figure 1(A)), and as a result, β-catenin phosphorylation was reduced and it became stable in the cytosol and efficiently translocated to the nucleus (Figure 1(A, B)). The 6-BIO treatment reactivated HIV-1 in ACH2 cells, as demonstrated by the significantly increased expressions of HIV-1 *gag* mRNA (Figure 1(C)). A dose-dependent manner for 6-BIO inactivating GSK3 and reactivating HIV-1 in ACH2 cells has been observed (Figure 1(A–C)). When ACH2 cells were treated with 6-BIO for different time, we found that the expression of β-catenin was increased, and the expression kept stable after 24 h stimulation (Figure 1(D)). HIV-1 reactivation from the treated cells has been observed over time (Figure 1(F)).

To confirm the observation is not an artefact in a single cell line, we performed the same experiment in HIV-1 latently infected J-Lat cells 10.2 and A2 clones[21], and a dose-dependent manner for 6-BIO inactivating GSK3 and reactivating HIV-1 has been observed (Figure 1(G, H)).

Lithium chloride (LiCl) is another well-known GSK3 kinase inhibitor by competition for magnesium, but LiCl does not alter GSK3 phosphorylation [25]. In consistence with 6-BIO results, LiCl treatment stabilized β-catenin, increased it nuclear translocation (Supplementary Figure 1 (A, B)), and reactivated HIV-1 in ACH2 cells (Supplementary Figure 1(C, D)).

Taken together, these results demonstrate that the pharmacological inactivation of GSK3 kinase activity reactivates HIV-1 from latently infected CD4^+^ T cells.

### 6-BIO-suppression of GSK3 activates the β-catenin/TCF1 axis to reactivate HIV-1 in CD4^+^ T cells

**Figure 2.**
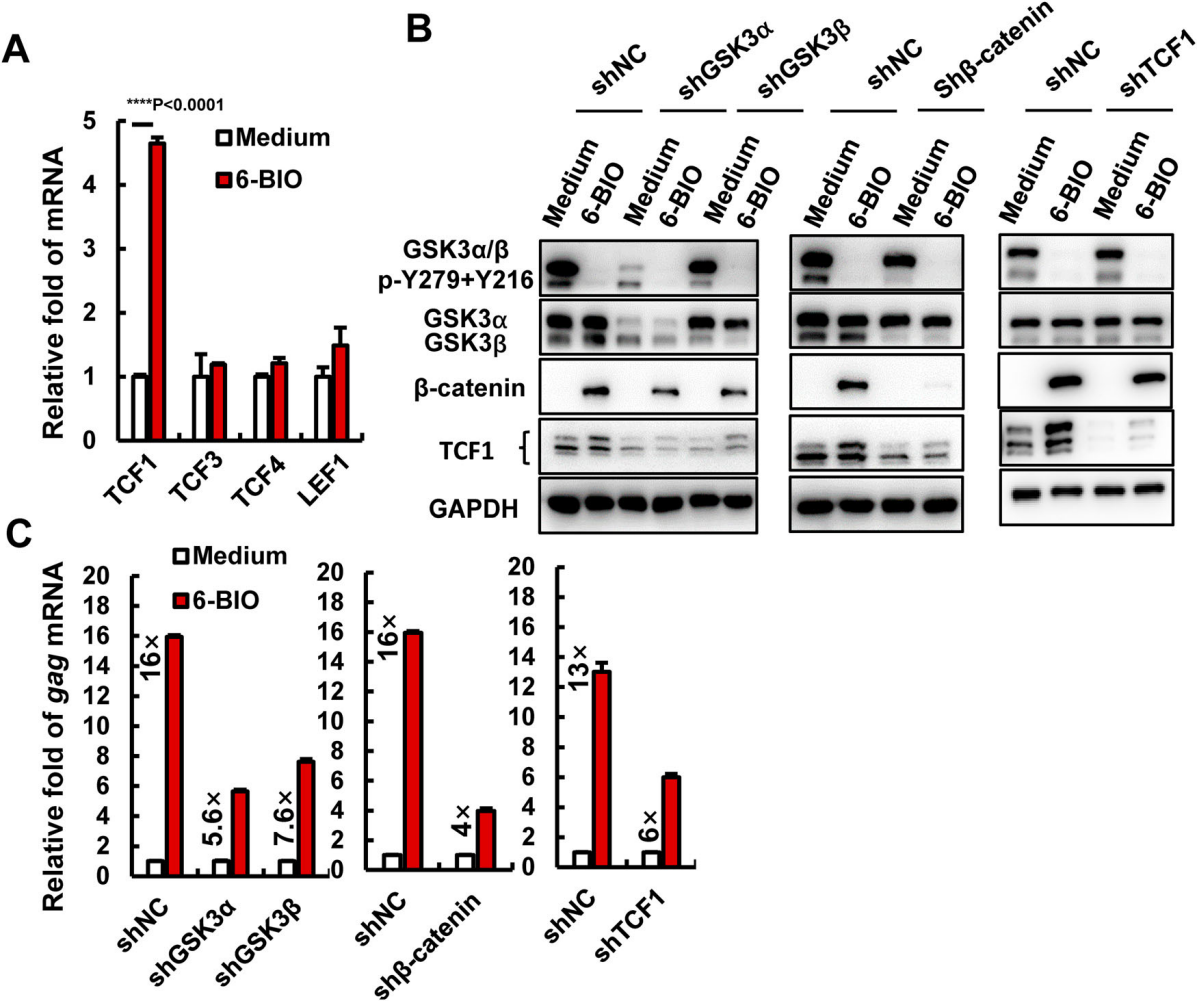
Inhibition of β-catenin/TCF1 axis attenuates 6-BIO-triggered HIV-1 reactivation. (A) The expression of TCF/LEF family members. ACH2 cells (1×10^6^) were treated with 6-BIO (1 μM) for 24 h, the expression of TCF1, TCF3, TCF4, and LEF1 was quantified by real-time qPCR and the relative enhancement fold was calculated. (B, C) Interference with the β-catenin/TCF1 pathway attenuates 6-BIO-induced viral reactivation. The endogenous GSK3α, GSK3β, β-catenin, or TCF1 was knocked-down with lentiviruses containing specific shRNAs for 48 h (B), and cells were further treated with 6-BIO (1 μM) for additional 24 h and viral reactivation was detected and calculated (C). Result is one representative from five independent repeats. Data are presented as mean ± SD. *** *P* < 0.001 denotes significant difference.

In the canonical Wnt signalling pathway, the nuclear β-catenin activates target genes through its interactions with transcription factors of TCF/LEF family members TCF1, TCF3, TCF4, and LEF1 [18–20]. 6-BIO treatment significantly up-regulated TCF1 expression, but not other members in ACH2 cells (Figure 2(A)), and 6-BIO-stimulated expression of TCF1 and nuclear location were observed (Figure 1(A, B, D, E, G)). Moreover, ACH2 shows the higher expressions of TCF1 and LEF1 (Supplementary Figure 2 (A), left panel). These data indicate that 6-BIO-induced HIV-1 reactivation might be achieved by activating β-catenin/TCF1 axis.

To ascertain this, we monitored the 6-BIO-induced HIV-1 reactivation when the endogenous expressions of GSK3α/β, β-catenin and TCF1 in ACH2 cells were knocked-down by infections with lentiviruses containing specific short hairpin RNAs (shRNA) (Figure 2(B)). In the shRNA negative control (shRNA), 6-BIO treatments induced 13- to 16-fold HIV-1 reactivation (Figure 2(C)), in contrast, the GSK3α- and GSK3β-knockdown reduced that to 5.6- and 7.6-fold viral reactivations, equivalent to 2.8- and 2.1-fold attenuation (Figure 2(C)). Similarly, the β-catenin knockdown attenuated 6-BIO-induced HIV-1 reactivation by 4-fold, and the TCF1 knockdown attenuated it by 2.2-fold (Figure 2(C)). To further confirm the essential role of β-catenin/TCF1 signalling in modulating HIV-1 reactivation, we used HIV-1 chronically infected human monocytic cell line U1 [26]. Unlike ACH2 cells, U1 showed a higher expression of TCF4, but not TCF1 or the other two members (Supplementary Figure 2 (A)). 6-BIO treatment did not upregulate the expressions of TCF1 or other members (Supplementary Figure 2(B)). Although 6-BIO treatment inhibited GSK3 kinase activity and increased β-catenin accumulation in U1 cells (Supplementary Figure 2(C)), as expected, the 6-BIO treatment did not induce HIV-1 reactivation in U1 cells (Supplementary Figure 2(D)). Taken together, these results demonstrate that 6-BIO-induced HIV-1 reactivation in CD4^+^ T cells is achieved by activating the β-catenin/TCF1 axis.

### Transcriptome analysis reveals 6-BIO-mediated activation of T cells and cellular β-catenin/TCF1 signalling

**Figure 3.**
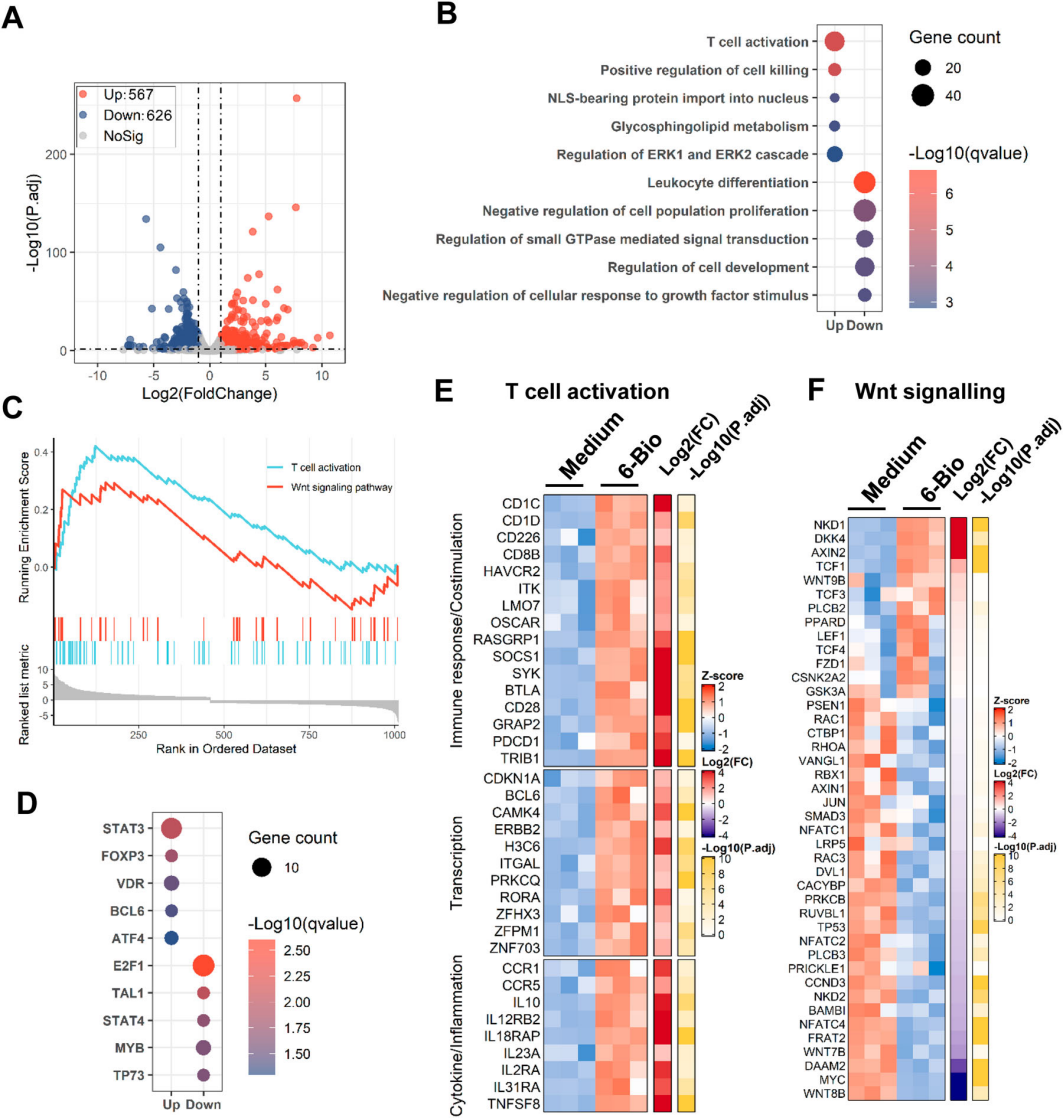
Transcriptome analysis reveals 6-BIO-mediated activation of T cells and cellular β-catenin/TCF1 signalling. ACH2 cells were treated with 6-BIO (1 μM) for 24 h, (A) Volcano plot of differentially expressed genes (DEGs) comparing 6-BIO-treated versus untreated (medium) cells. (B) Gene ontology (GO) functional enrichment analysis of DEGs. The colour bar indicates the minus logarithm of *q* values, and bubble size indicates the absolute gene counts enriched in a GO term. (C) GSEA showing the distribution of the gene sets that related to T cell activation and Wnt signalling pathway and the enrichment scores based on DEGs. (D) Transcription-factor enrichment analysis of DEGs. The colour bar indicates the minus logarithm of *q* values, and bubble size indicates the gene enrichment ratio regulated by a transcription factor. (E, F) Heatmaps showing relative expression level (left panel), fold change (middle panel), and adjusted *p* values (right panel) for gene sets related to T cells activation (E) and Wnt signalling (F).

Next, we performed transcriptome analysis to confirm 6-BIO-mediated activation of CD4^+^ T cells and cellular β-catenin/TCF1 signalling. ACH2 cells were treated with 6-BIO for 24 h, then the transcriptome was analysed using standard protocols. Data from three independent repeats were analysed. The volcano plot displayed a total of 567 up-regulated genes and 626 down-regulated genes after ACH2 cells were treated with 6-BIO (Figure 3(A)). Gene ontology (GO) functional enrichment analysis of differently expressed genes (DEGs) showed obvious upregulation of gene sets that regulate T cell activation, in contrast, the gene sets that regulate leukocytes differentiation, cell proliferation and development, and small GTPase-mediated signal transduction were down-regulated (Figure 3(B)). The GSEA linked the up-regulated genes to the regulation of T cell activation and Wnt signalling pathway (Figure 3(C)). Transcription-factor enrichment analysis of DEGs showed that the up-regulated transcription factors were mainly the signal transducer and transcription activators, such as *STAT3*, *FOXP3*, *VDR*, etc., and the down-regulated transcription factors were mainly linked to the regulations of cell proliferation and cell cycle, such as *E2F1*, *TAL1*, etc. (Figure 3(D)).

The core DEGs related to the regulation of T cell activation and Wnt signalling pathway were catalogized. The up-regulated genes regulating immune responses and transcriptions included *CD1C*, *SOCS1*, *SYK*, *BTLA*, *CD28*, *TRIB1*, *CAMK4*, *H3C6*, *RORA*, etc. (Figure 3(E)); the DEGs related to the regulation of inflammation and cytokine signalling mainly included *CCR1*, *IL-10*, *IL12RB2*, *IL18RAP*, *IL2RA*, *TNFSF8*, etc. (Figure 3(E)). Though 6-BIO treatment induced the expressions of Wnt signalling suppressors such as *NKD1*, *DKK4*, and *AXIN2* (Figure 3(F)), the upregulation of TCF1 has been observed upon 6-BIO treatment (Figure 3(F)). Taken together, the transcriptome analysis reveals 6-BIO-mediated activation of T cells and cellular β-catenin/TCF1 signalling.

### 6-BIO treatment enhances TCF1’s binding to 5′-LTR to promote HIV-1 transcription

**Figure 4.**
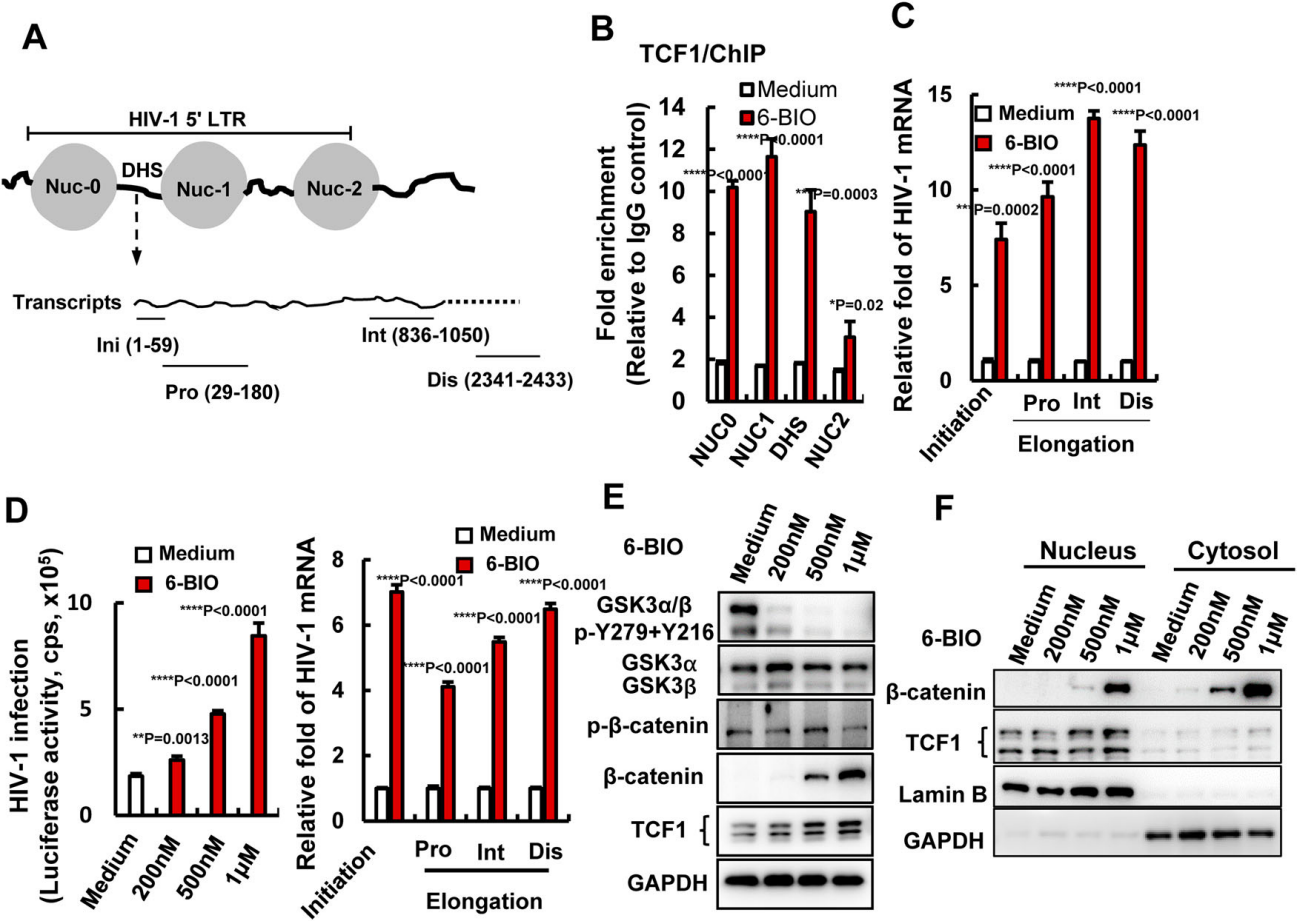
6-BIO treatment enhances TCF1’s binding to 5′-LTR to promote HIV-1 transcription. (A) A schematic diagram of HIV-1 5′-LTR and the primers used for quantifying the initiation and elongation of HIV-1 transcription in the products of transcribed viral mRNAs. Pro, proximal; Int, intermediate; Dis, distal. ACH2 cells was treated with 6-BIO (1 μM) for 24 h, (B) cells were subjected to ChIP assay to detect TCF1 binding to HIV-1 5′-LTR, and fragments of HIV-1 5′-LTR nucleosomes (NUC0, NUC1, NUC2) and the DHS were amplified by PCR and the fold enrichment was calculated, or (C) transcribed viral mRNAs were isolated and specific primers were used to quantify the initiation and elongation of HIV-1 transcription. (D–F) 6-BIO treatment promotes HIV-1 infection of Jurkat T cells and viral transcription. Jurkat T cells (1×10^6^) were acutely infected with pseudotyped HIV-Luc/NL-3 (5 ng p24^Gag^) for 24 h in presence of indicated concentrations of 6-BIO. Viral infection was measured by quantifying the luciferase activity, the initiation and elongation of HIV-1 transcription were measured (sample from 1 μM 6-BIO) (D), and the GSK3 activity, the expressions of β-catenin and TCF1 and their nuclear tranlocation were detected by Western blotting (E, F). Result is one representative from four independent repeats. Data are presented as mean ± SD. **P <0.01, ***P <0.001 and ****P < 0.0001 denote significant difference.

We next dissected the mechanism of 6-BIO-mediated activation of β-catenin/TCF1 signalling for reactivating HIV-1. The nuclear β-catenin binds to TCF1 and acts as a co-activator to enable the recognition of TCF1-specific sequence motifs in promoters and enhancers of Wnt targeted genes [27]. HIV-1 latency is mainly characterized by a reversible silencing of 5′-long-terminal repeat (LTR)-driven transcription of provirus. To explore whether 6-BIO treatment can promote TCF1 binding within the 5′-LTR promoter of HIV-1 proviral DNA, we performed ChIP assay in ACH2 cells, and found that 6-BIO treatment significantly promoted TCF1 binding to the positioned nucleosomes (NUC0, NUC1, NUC2) and the intervening enhancer regions DHS (DNase hypersensitive site) of 5′-LTR (Figure 4(A, B)). As the consequence, 6-BIO treatment significantly increased the initiation and elongation of HIV-1 5′-LTR-driven transcription (Figure 4(C)). The initiation and elongation of HIV-1 5′-LTR-driven transcription were investigated through monitoring (real-time PCR products with specific primers (Figure 4(A)) (Supplementary Table 1) [28,29].

To further confirm the regulation of 6-BIO-triggered β-catenin/TCF1 signalling on HIV-1 transcription, Jurkat T cells were acutely infected with pseudotyped single-cycle infectious HIV-Luc/NL-3 (CXCR4 tropic) for 24 h. A dose-dependent manner of enhancement of viral infection was observed upon 6-BIO treatment, and 6-BIO treatment significantly increased the initiation and elongation of HIV-1 5′-LTR-driven transcription (Figure 4(D)). Concurrently, 6-BIO treatment activated β-catenin/TCF1 signalling with a dose-dependent manner. GSK3 kinase activity in Jurkat T cells was inhibited by 6-BIO treatment as demonstrated by the diminished phosphorylation of both α and β isoforms of GSK3 at Tyr216 and Tyr 279 (Figure 4(E)), and as a result, β-catenin phosphorylation was reduced and it became stable in the cytosol and efficiently translocated to the nucleus (Figure 4(E, F)), and a dose-dependent upregulation of TCF1 expression and increased nuclear location were observed (Figure 4(E, F)). Taken together, these data demonstrated that 6-BIO treatment enhances β-catenin/TCF1 signalling and promotes HIV-1 transcription.

### 6-BIO treatment alters epigenetic modification of histones in HIV-1 5′-LTR promoter

Besides the direct promotion of viral transcription by binding to HIV-1 5′-LTR, TCF1 regulates gene expression can also be achieved through its ability to influence epigenetic modifications. The selective targeting of TCF1 in silent chromatin creates chromatin accessibility through epigenetically modifying histones by gaining active marks H3K27ac, H3K4me3, and erasing repressive marks H3K9me3, H3K27me3, and the ability of TCF1-mediated epigenetic modifications are known to regulate T cell development [30]. Notably, the above-mentioned repressive and activate epigenetic marks have also been found to guide chromatin-remodelling complex of HIV-1 5′-LTR promoter to modulate transcriptional activity of HIV-1 latency [4,31,32].

**Figure 5.**
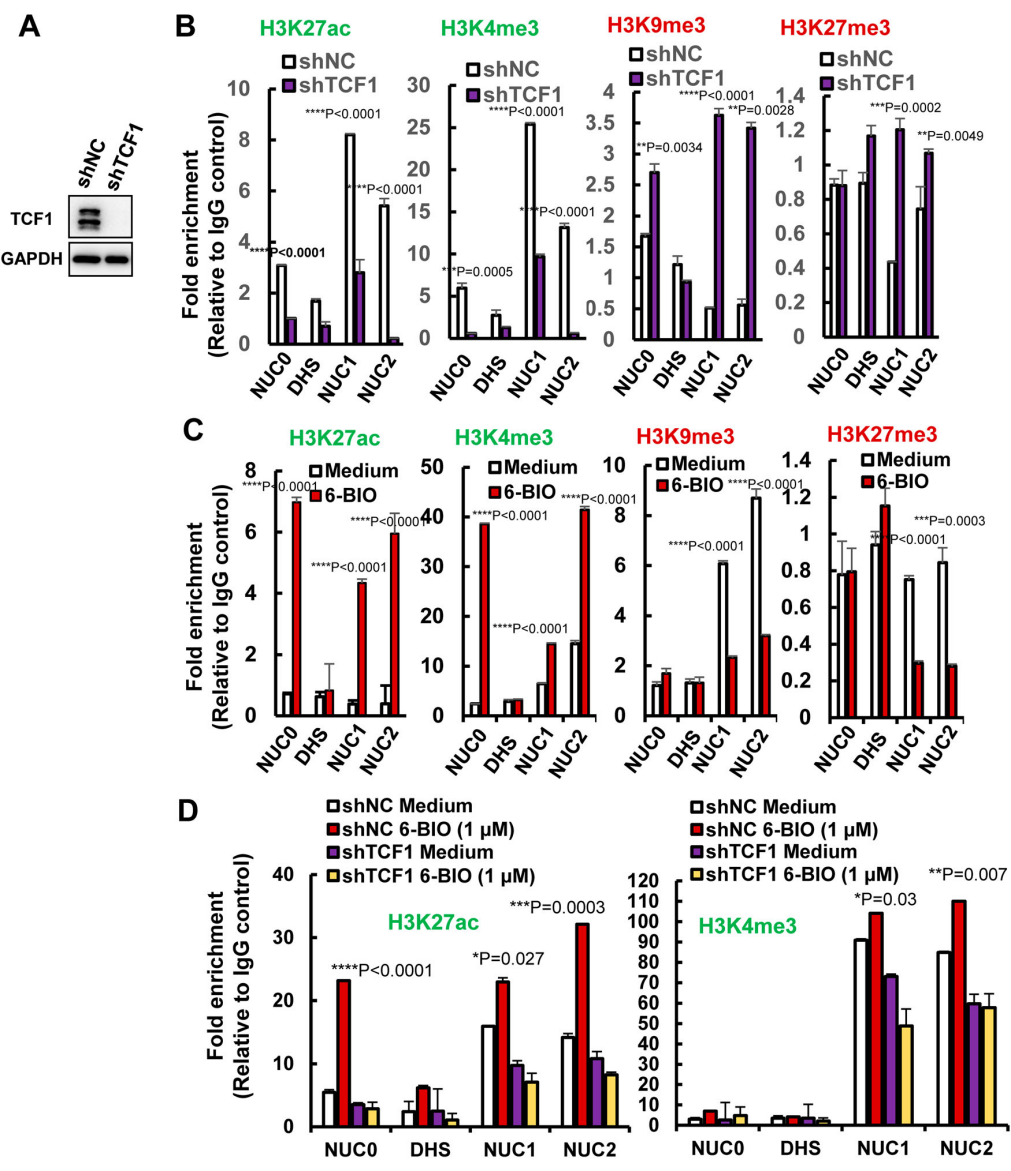
6-BIO treatment alters histone epigenetic modification of HIV-1 5′-LTR. (A, B) TCF1 knockdown alters histones epigenetic modification of nucleosomes in HIV-1 5′-LTR. The endogenous TCF1 in ACH2 cells was knocked-down with lentiviruses containing specific shRNAs for 48 h (A), and the epigenetic modifications of active marks H3K27ac, H3K3me3 and repressive marks H3K9me3, H3K27me3 in nucleosomes of HIV-1 5′-LTR were analysed by ChIP assay (B). (C, D) 6-BIO treatments alter histones epigenetic modification of HIV-1 5′-LTR. ACH2 cells without TCF1 knockdown (C) or with TCF1 knockdown (D) were treated with 6-BIO (1 μM) for 24 h and cells were subjected to ChIP assay. Result is one representative from four independent repeats. Data are presented as mean ± SD. **P <0.01, ***P <0.001 and ****P < 0.0001 denote significant difference.

We found that TCF1 modulated epigenetic modifications of histones in the HIV-1 5′-LTR promoter region. TCF1 knockdown in ACH2 cells (Figure 5(A)), decreased the active marks of H3K27ac and H3K4me3 in histones of Nuc0, Nuc1, and Nuc2, and increased the repressive marks of H3K9me3 and H3K27me3 on these histones (Figure 5(B)). Intriguingly, 6-BIO treatment significantly increased the active marks H3K27ac, H3K4me3 and reduced the repressive marks H3K9me3, H3K27me3 on these histones (Figure 5(C)). TCF1 knockdown abolished 6-BIO-triggered upregulation of H3K27ac and H3K4me3 (Figure 5(D)), indicating the requirement of TCF-1 in 6-BIO-induced epigenetic modifications of histones in the HIV-1 5′-LTR promoter. Taken together, these results demonstrate that 6-BIO treatment alters epigenetic modification of histones of HIV-1 5′-LTR, gaining the active form to initiate viral transcription.

### 6-BIO-triggered β-catenin/TCF1 signalling reactivates HIV-1 in cART-suppressed patient cells

**Figure 6.**
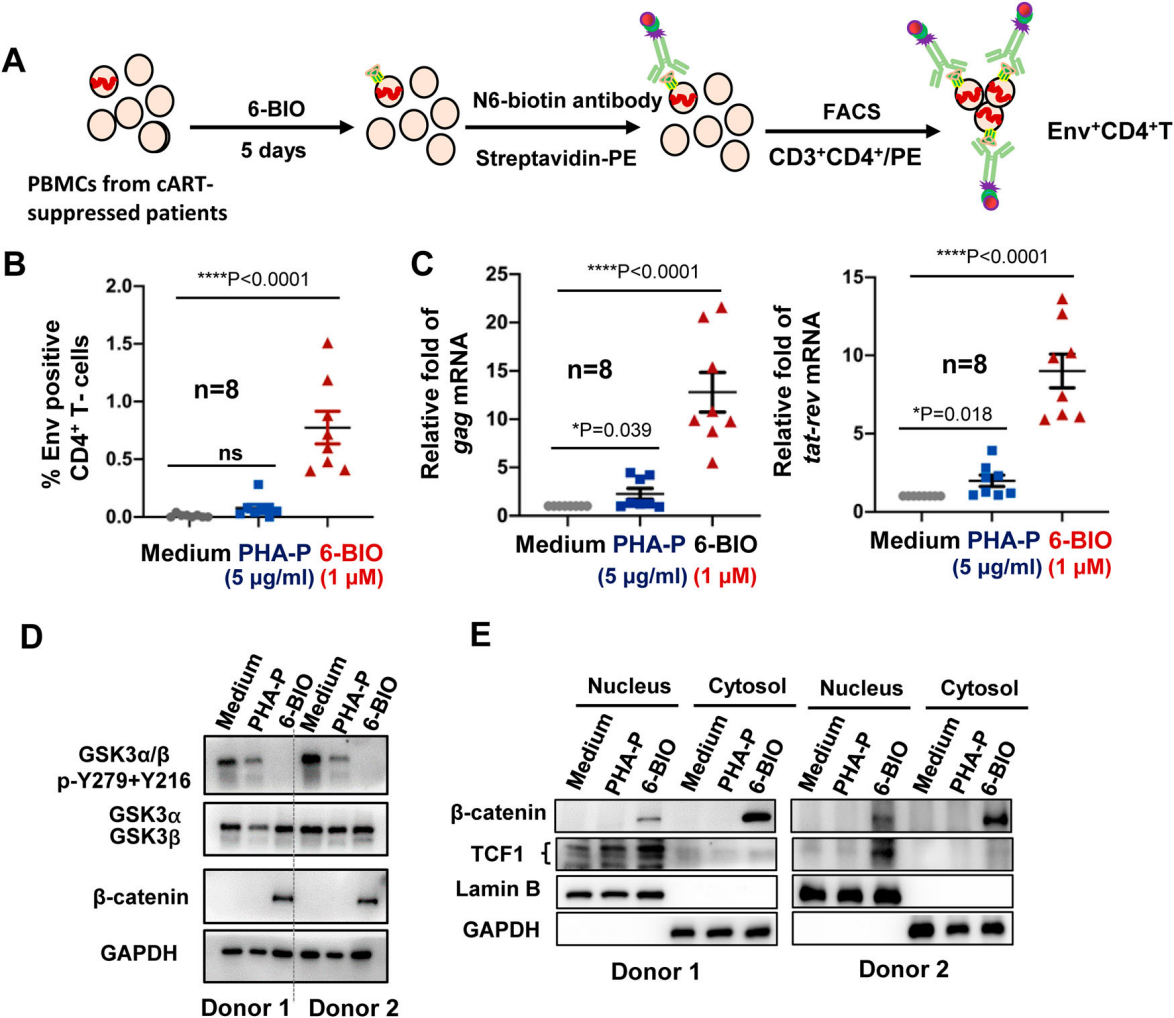
6-BIO-triggered activation of β-catenin/TCF1 axis reactivates HIV-1 in PBMCs isolated from cART-suppressed patients. PBMCs (1×10^7^) isolated from cART-suppressed HIV-1 patients were treated with 6-BIO (1 μM) or PHA-P (5 μg/ml) for 5 days, viral reactivation was measured by quantifying the percentage of Env^+^ CD4^+^ T cells (A, B), or by quantifying the production of intracellular *gag* or *tat*-*rev* mRNAs, and the enrichment fold for viral reactivation relative to medium treatment was calculated (C). (D, E) 6-BIO promotes β-catenin nuclear translocation in PBMCs from cART-suppressed HIV-1 patients. The PBMCs from 2 representative donors were harvested for subjecting immunoblotting, to detect the total levels and protein expression and phosphorylation (E), and to determine nuclear and cytoplasmic levels of β-catenin and TCF1. *P <0.05 and ****P <0.001 denote significant difference.

To confirm the role of 6-BIO-triggered activation of β-catenin/TCF1 pathway in reactivating HIV-1 observed using cell lines, we then tested 6-BIO treatment in primary PBMCs isolated from cART-suppressed patients (Supplementary Table 2). Phytohemagglutinin-P (PHA-P) treatment was used as the positive stimulation control. Viral reactivation was detected with flow cytometry to analyse the HIV-1 Env expression on CD4^+^ T cells (Figure 6(A)). Results showed that 6-BIO treatment significantly reactivated HIV-1 in patient CD4^+^ T cells (Figure 6(B)), and this was also confirmed by significantly increased quantity of cell-associated HIV-1 *gag* and *tat*-*rev* mRNA (Figure 6(C)). 6-BIO-mediated suppression of GSK3 kinase activity and the followed activation of β-catenin/TCF1 signalling were also confirmed in PBMCs from 2 cART-suppressed patients. The inhibition on GSK3 kinase activity was demonstrated by reduced phosphorylation of Tyr216 and Tyr 279 (Figure 6(D)), increased cytosolic stabilization and greater nuclear translocation of β-catenin (Figure 6(D, E)), and increased TCF1 nuclear location (Figure 6(E)). These results demonstrate that 6-BIO-induced suppression of GSK3 kinase activity and the followed activation of β-catenin/TCF1 axis reactivates HIV-1 in PBMCs isolated from cART-suppressed patients.

### 6-BIO reverses SIV latency in rhesus macaques

**Figure 7.**
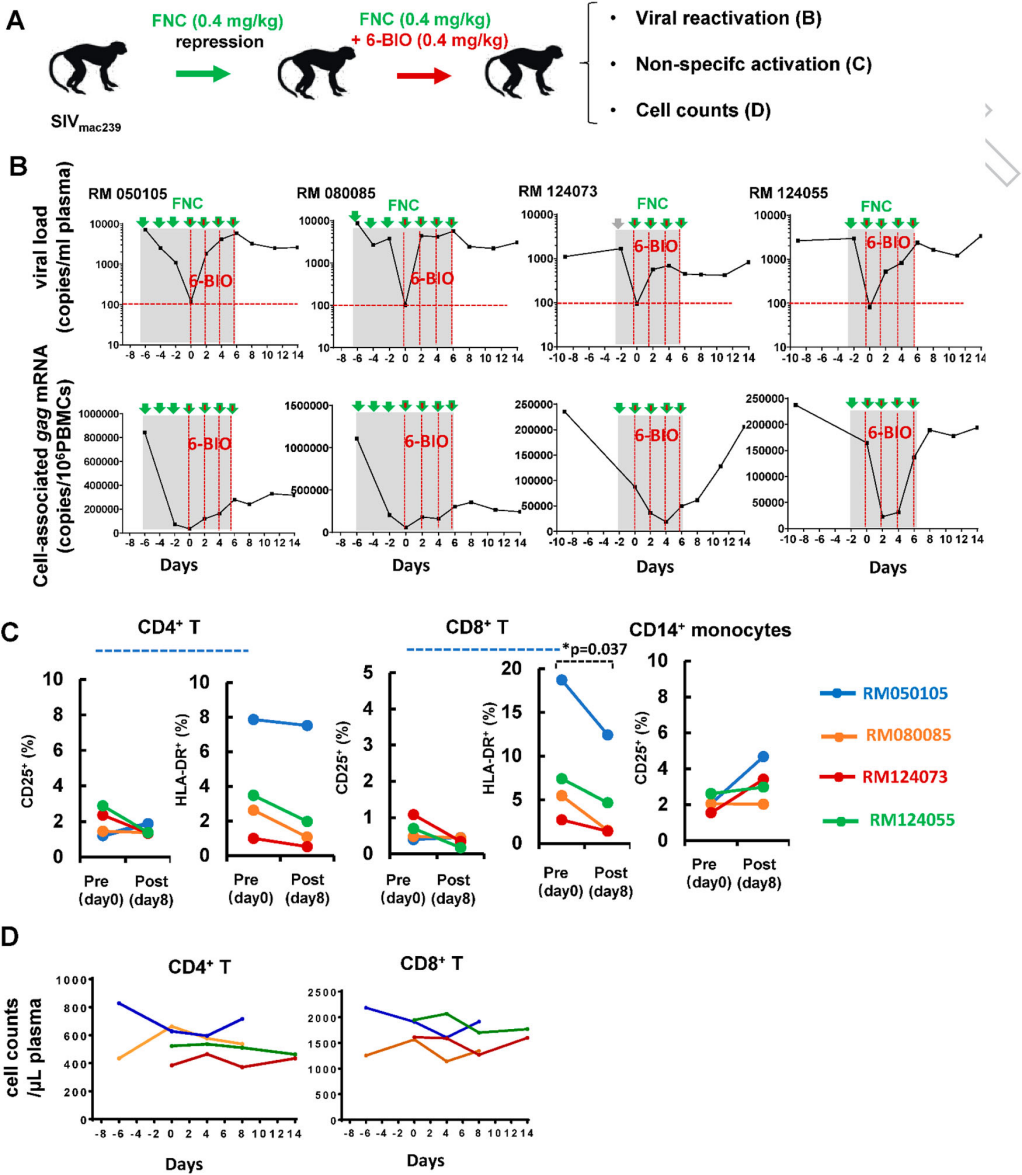
6-BIO reverses SIV latency in rhesus macaques. Four Chinese rhesus macaques were challenged with 5000TCID_50_ amounts of SIV_mac239_ intravenously via saphenous vein, and animals then were treated with FNC (0.4 mg/kg), followed with intravenous infusions of 0.4 mg/kg 6-BIO every two days (A). (B) Viral reactivation was measured by defining the production of both plasma and cell-associated *gag* mRNAs. (C) Non-specific activation assay. PBMCs were isolated at the time point of pre- (day 0) and post- (day 8) treatment with 6-BIO, and cells were immunostaining and the surface expressions of CD25 and/or HLD-DR on the CD4^+^ T-lymphocytes, CD8^+^ T-lymphocytes, and CD14^+^ monocytes were analysed with flow cytometry. (D) The plasma cell numbers of CD4^+^- and CD8^+^-T-lymphocytes were longitudinally monitored using BD TruCount tubes. *P <0.05 denotes significant difference.

To further investigate the role of 6-BIO in a more controlled setting, we next evaluated the latency reversal activity of 6-BIO in four Chinese-origin rhesus macaques. These macaques were challenged with 5000TCID_50_ of SIV_mac239_, and then were treated with a novel nucleoside reverse transcriptase inhibitor FNC (2’-deoxy-2’-β-fluoro-4’-azidocytidine) we developed (Chang J. et al., patents: ZL 200710137548.0, US 8835615 B2, EP 2177527 B1) (Figure 7(A)). FNC has superior anti-HIV-1 potency and long-lasting action and thus has been approved by the National Medical Products Administration of China for clinical treatment of HIV-1 patients in July 2021. FNC treatment (0.4 mg/kg) achieved the suppression of SIV viremia in all animals (below 100 copies/mL plasma) (Figure 7(B), upper panels). Intravenous infusions of 0.4 mg/kg 6-BIO were administered every two days. Viral reactivation, defined as the re-production of both plasma (Figure 7(B), upper panels) and cell-associated *gag* mRNAs (Figure 7(B), lower panels), was observed after the first dose and kept increase along with the further 6-BIO administration in all macaques. Non-specific activation of host cells can provide more susceptible target cells for HIV-1 infection [33]. The administration of 6-BIO treatment did not induce the non-specific activation of CD4^+^ T, CD8^+^ T, and CD14^+^ monocytes, as shown by no increased surface expressions of CD25 and/or HLD-DR (Figure 7(C)); moreover, 6-BIO did not lead to decline of the absolute number of CD4^+^ and CD8^+^ T cells over the period of treatment (Figure 7(D)). Taken together, these results demonstrate that 6-BIO efficiently induces SIV latency reversal in rhesus macaques.

## Discussion

The reversible repression of HIV-1 5′- LTR-mediated transcription represents the main mechanism for HIV-1 to maintain latency [1–3,6,8,9]. Identification of host factors/pathways that modulate LTR activity and viral latency helps develop new antiretroviral therapies. In this study, by targeting GSK3 for inhibition with small molecule agonists 6-BIO and LiCl, we provide evidence that activation of β-catenin/TCF-1 pathway reactivates HIV-1 from latently infected CD4^+^ T cells. Given 6-BIO has been proposed for treatments of a wide range of diseases [34–37], it provides a potent LRA candidate that can be used in combination with cell immunotherapies or antiretroviral drugs in the “Shock and Kill” strategy to eradicate HIV reservoirs.

The canonical Wnt/β-catenin pathway is evolutionarily conserved and plays important roles in stem cell proliferation and pluripotency, neurodevelopment, embryonic development, and tumorigenesis [38–41]. Dysregulation of Wnt/β-catenin signalling pathway has been associated with cancers and neurodegenerative diseases, and small molecules targeting Wnt/β-catenin pathway have been tested as potential drugs for treating these diseases [41–44]. Of note, Wnt/β-catenin signalling modulates the proliferation of CD4^+^ T cells that form long-lived HIV-1 reservoir. Blockade of the interaction between β-catenin and co-activator CREB-binding protein, by a Wnt/β-catenin pathway small molecule inhibitor PRI-724, decreases the proliferation of stem cell memory- and central memory-CD4^+^ T in ART-suppressed SIV_mac251_-infected rhesus macaques, but unable to not significantly reduce the viral reservoir size [45].

The expression and function of the downstream TCF/LEF family members may have the cell-subtype selection. Most studies of Wnt/β-catenin signalling in relation to HIV-1 infection have focused on the regulation of TCF/LEF family member TCF4, which has been identified as a repressor of HIV-1 replication in astrocytes and monocytes [46–50]. In astrocytes and monocytes, the activation of Wnt/β-catenin/TCF4 signalling restricts HIV-1 replication [46–52]. 6-BIO-conferred restriction of HIV-1 Tat-mediated transcription in astrocytes may be due to the alternative activation of β-catenin/TCF4 signalling [53]. HIV-1 5′- LTR promoter contains the specific binding sites for both TCF1 and LEF that have been demonstrated to positively regulate HIV-1 transcription [54,55]. In our study, the CD4^+^ T ACH2 cells show the higher expression of TCF1 and LEF1, therefore 6-BIO stimulates the β-catenin/TCF1 signalling for viral reactivation.

Transcriptome data also confirm 6-BIO-mediated activation of β-catenin/TCF1 signalling in CD4^+^ T cells ACH2. The core DEGs related to the regulation of inflammation and cytokine signalling mainly included *CCR1*, *IL-10*, *IL12RB2*, *IL18RAP*, *IL2RA*, *TNFSF8*, etc. Non-specific activation of host cells can provide more susceptible target cells for HIV-1 infection [33]. It seems that 6-BIO does not induce the non-specific activation of these HIV-1 target cells, at least in our study.

Our study has some limitations. 6-BIO evaluation was performed in the limited number of 4 SIV-infected monkeys; the 6-BIO reactivation was started right after the SIV suppression in monkeys, and a longer duration for SIV suppression is more appropriate in reflecting HIV-1 latency in the long-term cART-suppressed patients. Besides of the monkey/SIV models, the humanized mouse models provide alternative tools for fully evaluating 6-BIO activity.

Taken together, by using HIV-1 latently infected CD4^+^ T cell lines, resting CD4^+^ T cells from cART-suppressed patients and SIV-infected rhesus macaques, we demonstrate that the pharmacological suppression GSK3 kinase activity by 6-BIO reactivates HIV-1 from latently infected CD4^+^ T cells. These findings advance our understanding of the mechanisms responsible for viral latency, and provide the potent LRA that can be further used in conjunction of immunotherapies to eradicate viral reservoirs.

## Supplementary Material

Supplemental Material
