# Supplementary material for: Pharmacological suppression of glycogen synthase kinase-3 reactivates HIV-1 from latency via activating Wnt/β-catenin/TCF1 axis in CD4+ T cells
Source: Emerg Microbes Infect. 2022 Feb 1;11(1):391–405. doi: 10.1080/22221751.2022.2026198 (PMC8812804; doi:10.1080/22221751.2022.2026198)
Supplement: Supplemental Material [file TEMI_A_2026198_SM6888.zip › Suppl files/Supplementary information.docx]

**Pharmacological suppression of glycogen synthase kinase-3 reactivates HIV-1 from latency via activating Wnt/β-catenin/TCF1 axis in CD4^+^ T cells**

**Figure legends**

**Supplementary Figure 1. Pharmacological suppression of GSK3 kinase activity reactivates HIV-1 from ACH2 cells.** ACH2 cells (1×10^6^) were incubated with 6-BIO (1 μM) or LiCl (25 mM) for 24 h, (A, B) GSK3 kinase activity, and β-catenin and its subsequent nuclear translocation, were detected by Western blotting with specific antibodies; HIV-1 reactivation was measured by quantifying the production of *gag* mRNA (C), or by measuring the production of infectious viruses in the cell cultural supernatants as quantified by titration in TZM-bl indicator cells (D).

**Supplementary Figure 2.** (A) The expressions of TCF1, TCF3, TCF4 and LEF1 in ACH2 and U1 cells were quantified by Real-time (RT-) qPCR, and the expression was normalized with *gapdh*. U1 cells (1×10^6^) was treated with 6-BIO (1 μM) for 24 h, (B) the expression of TCF1, TCF3, TCF4 and LEF1 was quantified by Real-time (RT-) qPCR and the enhancement fold relative to medium treatment was calculated, (C) the expressions of GSK3, β-catenin and TCF1 and/or their phosphorylated forms were detected by Western blotting with specific antibodies; the production of HIV-1 Gag was also detected, and (D) HIV-1 reactivation was measured by quantifying the production of *gag* mRNA and the relative increase for *gag* mRNA level was calculated. Result is one representative from three independent repeats. Data are presented as mean ± SD. Two-tailed, unpaired, *t*-test was used to analyze the significant difference.

**Supplemental Table 1. Primers for (RT-) PCR**

**Supplementary Table 2. Patient information**
