# Supplementary figures and images for: Pharmacological suppression of glycogen synthase kinase-3 reactivates HIV-1 from latency via activating Wnt/β-catenin/TCF1 axis in CD4+ T cells
Source: Emerg Microbes Infect. 2022 Feb 1;11(1):391–405. doi: 10.1080/22221751.2022.2026198 (PMC8812804; doi:10.1080/22221751.2022.2026198)

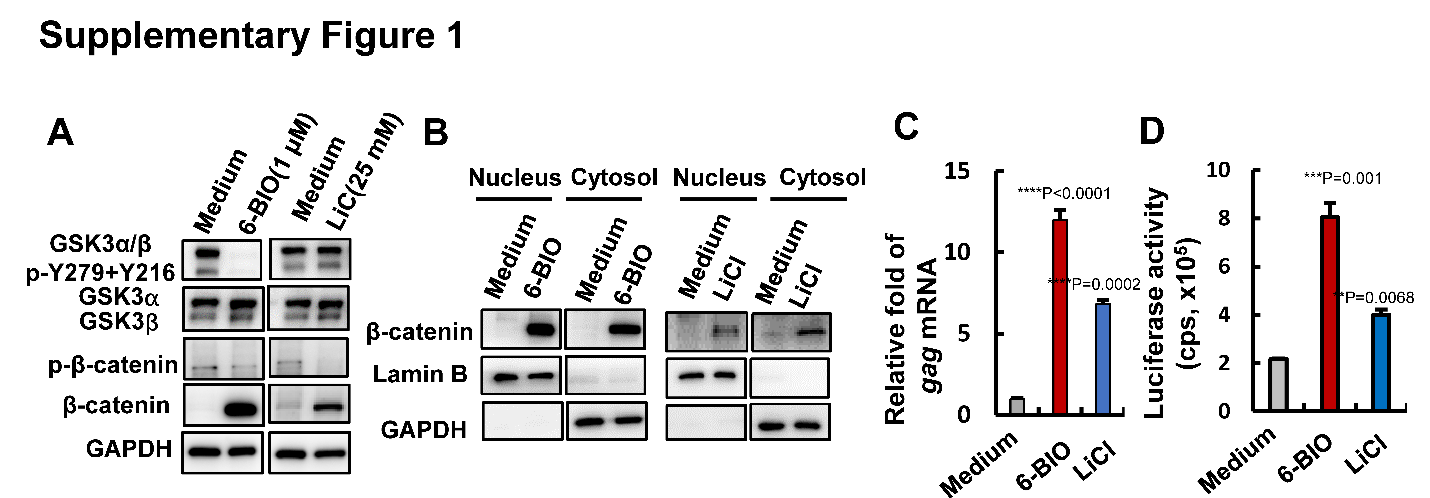


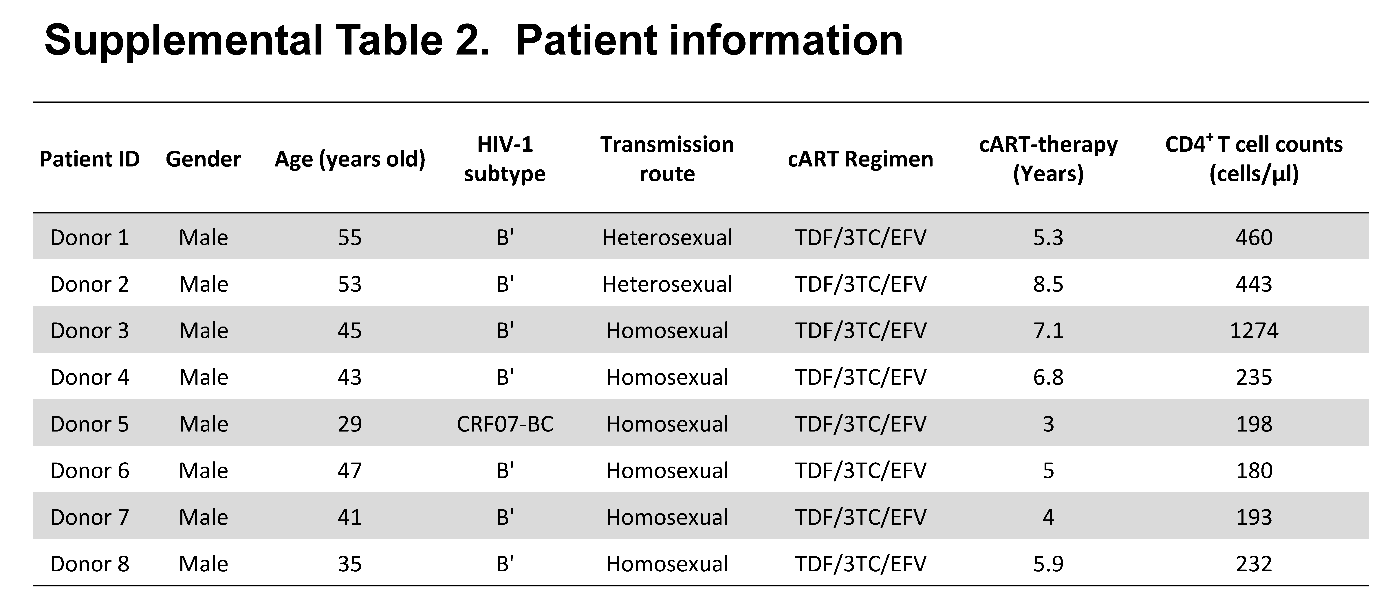

Supplement: Supplemental Material [file TEMI_A_2026198_SM6888.zip › Suppl files/Supll files.docx]

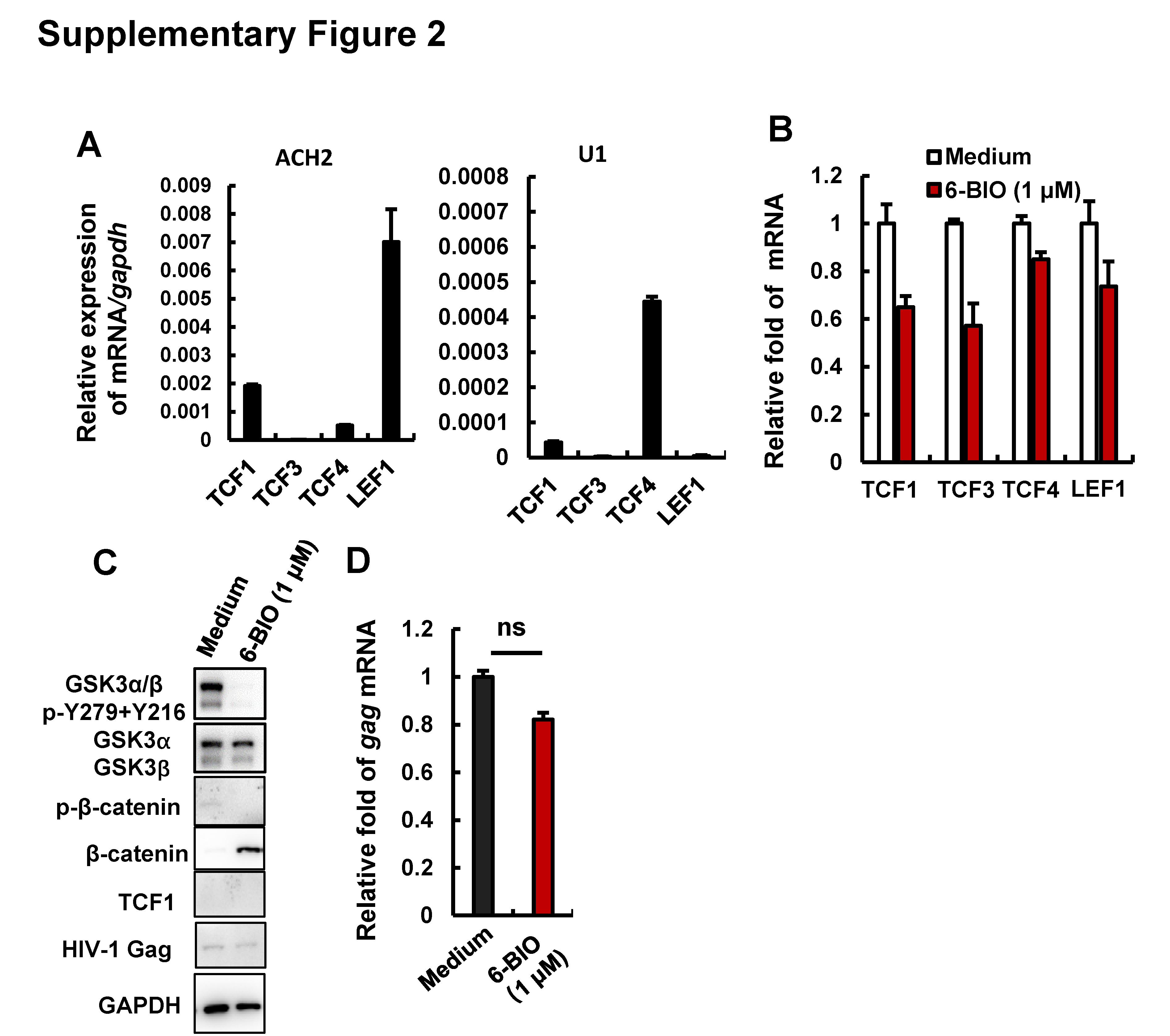

Supplement: Supplemental Material [file TEMI_A_2026198_SM6888.zip › Suppl files/Supplementary Figue 2.tif]

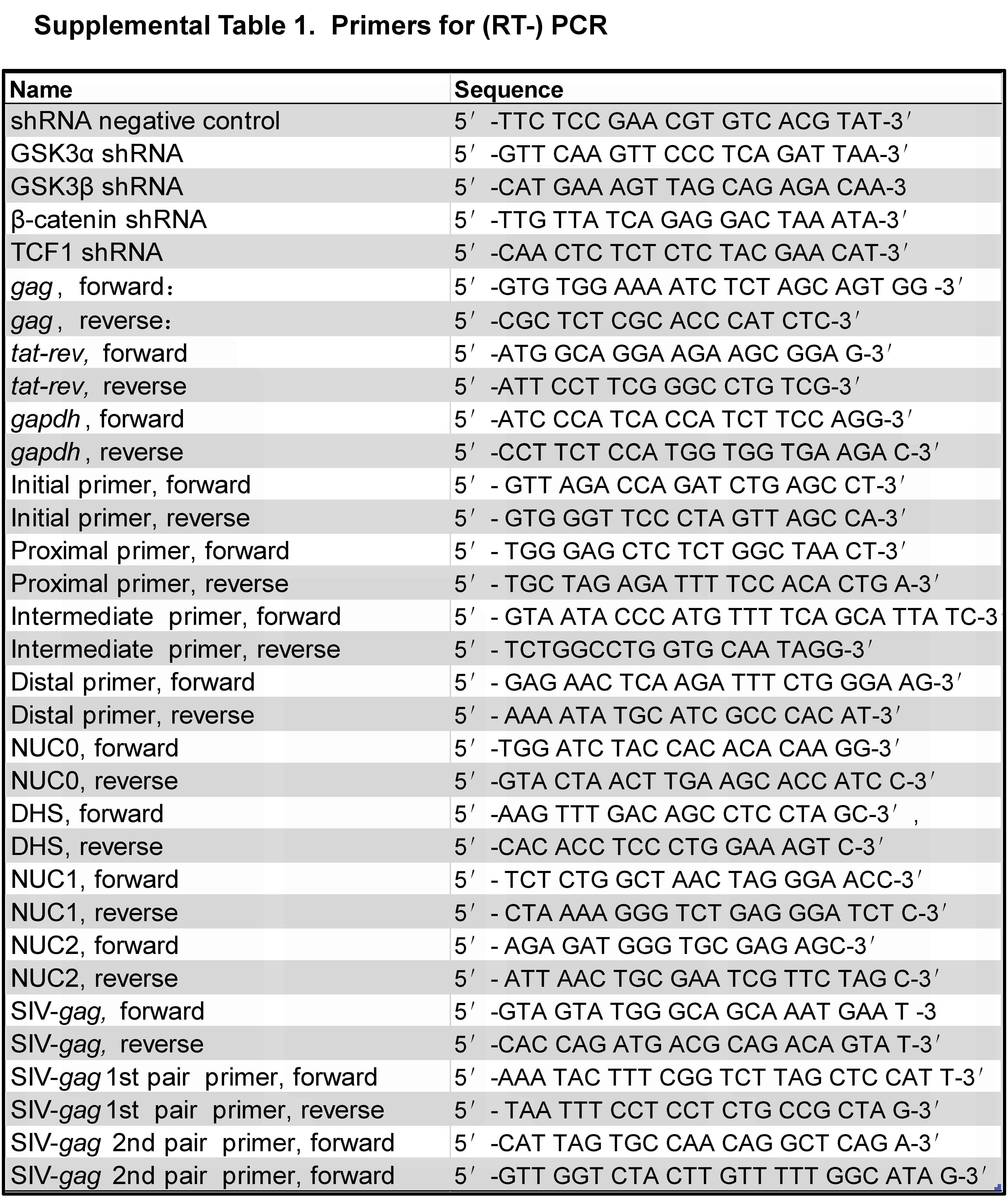

Supplement: Supplemental Material [file TEMI_A_2026198_SM6888.zip › Suppl files/Supplementary Table 1.tif]
